# Nurse experience during the COVID-19 pandemic: Implications for nurse leaders

**DOI:** 10.1097/01.NUMA.0000829268.46685.bb

**Published:** 2022-04-27

**Authors:** Elizabeth Roe, Sally Decker, Kristine Marks, Joyce Cook, Kourtney Garno, Julie Newton, Roberta Thrush

**Affiliations:** At Saginaw Valley State University in University Center, Mich., **Elizabeth Roe** and **Sally Decker** are professors of nursing. At MyMichigan Health in Midland, Mich., **Kristine Marks** is a nursing professional development specialist RN; **Joyce Cook** is a senior simulation specialist; **Kourtney Garno** is a unit manager; **Julie Newton** is an infection prevention specialist; and **Roberta Thrush** is a clinical research nurse.

## Abstract

Results of a two-question survey of nurses at one hospital identified 5 clear themes related to nurses' experiences during the pandemic and 7 areas for improvement, providing potential strategies for nurse leaders.

**Figure:**
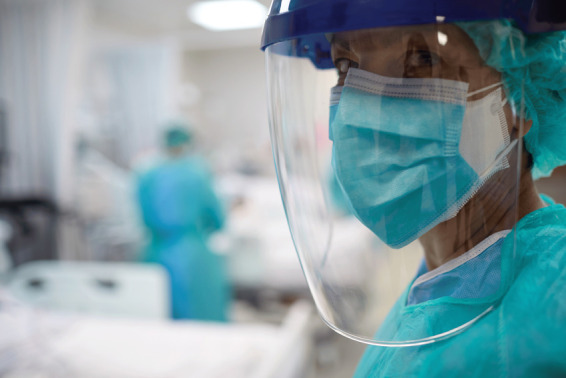
No caption available.

During the COVID-19 pandemic, nurses have been the provider with the most prolonged face-to-face contact with patients and associated exposure to patient suffering and viral transmission.^1^ Nurses have experienced practice changes, fear for themselves and their families, and moral distress from the inability to provide optimal care.^2^-^4^ Throughout this time, it has been important for nurse leaders to provide the needed support to nurses.

Leadership embodies three areas: leading the self, leading others, and leading the organization.^5^ During the pandemic, leaders have been responsible for decisions affecting both patients and staff. One important role of a nurse leader is understanding the needs of nursing staff.^6^ Balancing the needs of the staff and patients while considering quality metrics, data, and finances has been a challenge during the pandemic. Providing staff support fosters a positive workplace culture that results in increased patient satisfaction and staff well-being.^7^ With the exhaustion of resources, nurse leaders need to use the wisdom of clinical nurses in decision-making to preserve trusting relationships. During a crisis, decision-making tends to be more authoritarian, and the goals of person-centered care are difficult to maintain.^8^ Social media and the sensationalism of the pandemic have added to the crisis and called for nurse leaders to understand the needs of clinical nurses.^9^

The 2020 WHO report “State of the World's Nursing 2020” stresses the importance of leadership to quality care and safety. Nurse leaders have been scrutinized during the COVID-19 pandemic, with a call for leaders to be visible and active participants in the decisions that shape the healthcare system's response.^8^,^10^ Studies early in the COVID-19 pandemic documented the workplace as a source of stress for nurses and emphasized the importance of leader support.^4^,^11^,^12^ Although many issues were present before the pandemic, the stress that the pandemic has put on the healthcare system has affected nurses dramatically. As part of a continuous improvement process at one hospital system, staff survey responses were solicited to help leaders be responsive to the nurses. This article discusses the results of that survey and the implications for nurse leaders.

## Background

In a case study of nursing leadership during the COVID-19 pandemic, Quinn and colleagues concluded the core of effective leadership is the ability to communicate with compassion and a willingness to serve.^13^ Recommendations for leaders during the pandemic have included using communication that's truthful, mindful, and relevant to practice.^14^ Rosa and colleagues recommended that leaders round regularly and include nurses in decisions, and Markey and colleagues encouraged nurse leaders to maintain ethical vigilance and find ways to empower, support, and enable nurses to apply ethical standards.^15^,^16^

Across countries and methods, researchers have documented the effects of pandemics on nurses. A review of studies on the psychological effects on clinicians working during viral outbreaks that included 59 studies from SARS, COVID-19, MERS, Ebola, H1N1, and influenza A virus subtype H7N9 indicated that staff members who had contact with infected patients had more acute and posttraumatic stress and psychological distress.^17^ Clear communication, access to proper personal protective equipment (PPE), adequate rest, and practical and psychological support were associated with reduced morbidity.^17^

Since the start of the COVID-19 pandemic, research from across the world has consistently documented how the pandemic has negatively impacted nurses. One study of 325 nurses in the Philippines found that 37% had dysfunctional levels of anxiety.^18^ A survey of nurses in 42 hospitals across China found that 9.4% of the nurses had depressive symptoms, 8.1% reported clinically significant anxiety, 6.5% had suicidal ideation, and 42.7% had somatic symptoms.^19^ A study of 705 nurses in Turkey found the average stress score indicated high perceived stress, burnout, and moderate depression.^20^ A survey of 3,676 nurses in Canada found that 47% experienced PTSD, 38% anxiety, 41% depression, and 60% high emotional exhaustion.^12^ In the US, similar experiences have been documented. In a survey that included 974 physicians, advanced practice providers, residents/fellows, and nurses in New York in April 2020, 57% experienced acute stress, 48% depression, and 33% had anxiety symptoms.^21^ Another study of 22 nurses interviewed in May and June of 2020 found the major theme of an emotional roller coaster, with nurses experiencing a range of intense emotions, including fear, anger, and exhaustion.^22^ In a survey of critical care nurses in the US with 285 responses collected from October 2020 through January 2021, participants reported a variety of negative physical and emotional symptoms.^3^

## Theory

This quality improvement project aimed to understand the experiences of nurses working at one hospital system during the COVID-19 pandemic using fundamentals from the relationship-based care (RBC) model and a qualitative approach. The RBC model provides both the philosophical foundation and practical infrastructure for the hospital system, which includes seven hospitals (rural and nonrural), physicians' offices, urgent care, and home care.^23^ RBC culture has the patient and family at the core and is committed to three key relationships—self, colleagues/coworkers, and patients/families—and six dimensions essential to the implementation of RBC, which include leadership, teamwork, professional nursing practice, patient-care delivery, resource-driven practice, and outcomes measurement.

## Methods

### Sample

A qualitative design was used to document nurses' experiences from their perspectives during the pandemic. The project was ruled exempt by both the university and hospital Institutional Review Boards. Nurses were asked to respond to two open-ended questions about their experiences during the pandemic: 1. What has it been like to be a nurse at (agency) during the COVID-19 pandemic? 2. What might be more helpful or useful to nurses at (agency) during the pandemic?

A total of 1,632 nurses were asked to participate by the hospital's Nursing Research Council, and 476 completed both sections of the instrument for a response rate of 29%.

### Data collection

The survey was distributed via email and was available for 2 weeks in February 2021. After data were collected, responses were transferred to a word processing program and a team of researchers (two nursing faculty members and four members from the agency serving in various educational and clinical roles) completed content analysis.

### Thematic analyses

**Question one: Nurse experience**. First-level coding was completed using the organization's RBC model. Professional practice and care delivery were combined into one code, and outcomes included nurse and patient outcomes. Both physical and staffing resources were considered, and leadership included aspects of communication and management. Two faculty members independently completed first-level coding, identifying meaning units (sentence fragments) and creating coding rules. The practice partners then reviewed the meaning units with associated codes to determine if the statements fit the codes from a contextual perspective and the use of the RBC model in practice. Following review by the practice partners, researchers reached an agreement regarding first-level coding.

In second-level coding, investigators used the five coded sections to identify subcategories. The initial codes of outcomes and leadership had the most responses and subcategories. Again, faculty investigators identified subcategories and verified the categories and meaning units with agency investigators.

In the next step, the subcategories were collapsed to create themes. This was accomplished by the entire investigative team in a virtual meeting. As a final step, the group used the themes to back-code to the meaning units and identified exemplars for the themes. All researchers reached an agreement on the back-coding for the exemplars.

**Question two: Strategies for improvement**. Following completion of the analysis of the first open-ended question, investigators used the same method for the second open-ended question.

## Results

**Question one**. The concepts from the RBC model were used to identify the subcategories and themes. Researchers derived five themes from the data: 1. Different (polarized) perceptions of leadership/communication with varied connection to levels of leadership; 2. Balance of meeting patient and nurse needs and managing exhaustion; 3. Rapid practice changes and doing things differently, which varied with unit and role; 4. Increase in need/change of resources (PPE, staffing) requiring teamwork; and 5. Emotional experience (stressful/scary). Table 1 includes the first-level codes from the RBC Model, subcategories, and themes with exemplar meaning units from the interviews.

**Table 1:** Coding for question one (What has it been like to be a nurse during the COVID-19 pandemic?)

First-level code	Subcategories	Themes	Exemplar meaning units
Leadership	Frequent changes in policies Visitor policy (communication and implementation) Communication frequent but confusing Inadequate staffing (differed by unit) Supportive first-level leadership Nonappreciative leadership Lack of training/preparedness PTO policy	Differing (polarized) perception of leadership/communication, varied connection with levels of leadership	“I felt as if my organization was proactive in trying to keep up with best practices and communication.” (Participant #169) “Changes didn't always involve bedside staff.” (Participant #364)
Teamwork	Team building Appreciative of team Mostly nurses as team Always a few negative team members	Balance of meeting patient and nurse needs and managing exhaustion	“The focus of patient-centered care shifted, and I feel like an assembly line worker.” (Participant #156) “Balance patient versus staff needs” (Participant #157)
Professional practice	Changes in practice/patient care (time, energy, communication related to PPE, acuity, and resources) Frequent changes in practice with rapidly changing requirements Balance between patient and nurse/staff safety Feeling unprepared to provide care for patients with COVID-19 Visitor policy impacted practice Rewarding	Rapid practice changes and doing things differently, which varied with role and unit	“Learning how to adapt to a quickly changing workplace – everything changed” (Participant #445) “Adapting to the day-to-day changes was stressful.” (Participant #468)
Resources	Lack of PPE (varied by role, unit) Staff shortage (varied by role, unit)	Increase in need/change of resources (PPE, staffing) requiring teamwork	“Donning and doffing all the PPE is very time-consuming.” (Participant #98) “Staff has helped each other, stood behind each other, and watched each other. We have all worked as a team.” (Participant #101)
Outcome	Mental health: stressful, scary/fearful; physical health: exhausting Overwhelming/chaotic/uncertain Differed by unit/role Challenging (required nurse resources but rewarding)	Emotional experience (stressful/scary)	“Every emotion has been experienced, from fear, exhaustion, and frustration to hope, excitement, and being proud of what I do and where I work.” (Participant #389)

**Question two**. Seven strategy areas were identified from the data related to the question about what would be more helpful to nurses: 1. Nurses' input/presence in decision-making; 2. Adequacy of preparedness and training (education, PPE, staffing); 3. Support of staff physically (breaks, better treatment when sick, mental health days) and emotionally (empathy, compassion from leadership, employee health, debriefing, stress reduction); 4. Visibility of management (focus on support, empathy, appreciation); 5. Consistent, accessible, relevant, prompt communication (vary depending on recipient); 6. Response to changes needed relating to COVID-19 practice demands (different staffing models, appropriate staff, different definitions of productivity); and 7. Compensation (paid time off [PTO], hazard pay). See Table 2 for improvement areas and exemplar meaning units for the second question.

**Table 2:** Coding for question two (What might be more helpful or useful to nurses during the pandemic?)

Improvement strategy areas	Exemplar meaning units
Nurse input/presence in decision-making	“Utilizing frontline workers to help generate policy” (Participant #125) “Bedside nurses were never asked opinions.” (Participant #134)
Adequacy of preparedness and training	“We hadn't been given extra training to care for COVID.” (Participant #21) “Cross-training nurses to help out in other areas” (Participant #133) “Continued education on testing guidelines” (Participant #213) “Staffing levels adjusted for COVID patients. Meeting staffing demands has been extremely hard on nurses.” (Participant #68)
Support of staff	“Education on coping” (Participant #407) “More compassion; more support from management” (Participant #64) “Value employees” (Participant #426)
Visibility of management	“More visibility of upper management” (Participant #390) “Increased kindness” (Participant #216) “For upper management to listen to staffing concerns” (Participant #191)
Consistent, accessible, relevant, prompt communication	“Communication being clear and concise. Getting right to the point.” (Participant #290) “Better communication of changes.” (Participant #132)
COVID-19 practice demands	“Staffing with nurses capable of doing job” (Participant #315) “Staffing levels adjusted for COVID patients.” (Participant #68)
Compensation	“Allowing nurses to take their much-needed PTO to get a mental and physical break” (Participant #351) “Hazard pay” (mentioned 27 times)

## Discussion

**Theme one: Differing perceptions of leadership**. Leadership support is important during a pandemic. In a study of 451 RNs in five hospitals in China, Zhao and colleagues found that inclusive leadership had an inverse relationship with psychological distress.^24^ For the hospital described in this article, the leadership strategies in place included frequent communication by various means, but the staff wanted leadership to be “more present,” preferably face-to-face and during times when they could have more contact with a diverse group of nurses (nights, weekends). Although this organization had several methods for communicating, some nurses desired more concise communication. The nurses surveyed recommended using regular parts of care provision, such as pop-ups on the electronic health record (EHR) or daily huddles for communication.

Staffing was another issue related to this theme. Although the agency was using alternative staffing models, survey respondents suggested more staff involvement and clarification of staffing ratios. In a study of nurse managers during the COVID-19 pandemic in the US, White found that leadership challenges included providing a different kind of support, revamping their approach, and addressing staff resistance and fears.^25^ In a study of primary health nurses during the COVID-19 pandemic in Australia, Halcomb and colleagues found that the nurses needed high-level communication support. Involving staff in decisions and problem-solving could be helpful to bridge these challenges for leadership.^26^ A study in the US found that nurses perceived inadequate leadership support and inequity within the healthcare team.^3^

**Theme two: Balance of meeting patient and nurse needs and managing exhaustion**. The agency described in this article used numerous support strategies including employee assistance for childcare and stress reduction programs. Suggestions for improvements included team-building activities across the agency; those provided were often confined to specific units. For example, one unit that was chosen to be a COVID unit in one of the hospitals used a creative approach. They renamed their unit the “COVID Cove” and decorated in a beach theme that was welcoming to both staff and patients. Other suggestions included using a variety of support strategies and focusing on interprofessional team building. There was some confusion about how available PTO could be used. Help with scripting and enforcement limitations on visitation policies were suggested. Because exhaustion was amplified by fear, participants indicated that strategies to deal with fear were also important. Balancing the needs of the patient and the nurse led to exhaustion. Similarly, in a qualitative study of healthcare providers across China, Liu and colleagues found that providers were challenged by working in a new context and reported exhaustion related to heavy workloads and protective gear.^27^ A study in the US also documented emotional and physical exhaustion.^22^

**Theme three: Rapid practice changes and doing things differently, varying with role and unit**. There were many practice changes related to the novelty and complexity of caring for patients with COVID-19, including the use of PPE, new medications and interventions, expanded roles for nurses, working more closely with other professions, and changes in visitor rules. This was similar to results from a study in Canada where nurses found challenges providing good care in response to practice changes and tensions from juggling patient care with other responsibilities.^4^ Although the organization offered resources in a variety of formats for addressing these challenges, participants were overwhelmed by all that had to be learned. It's important to explain policies and why they're changing. In addition, streamlining education and communication is essential. Specific and accurate information is needed, but it has to be presented in a concise, accessible way.

In an integrated review of qualitative studies of nurses' experiences working in hospital settings during a respiratory pandemic, Fernandez-Castillo and colleagues found one theme was that healthcare providers were challenged by working in a new context with heavy workloads and feeling powerless to handle the patients' conditions.^28^

**Theme four: Increase in need/change of resources (PPE, staffing) requiring teamwork**. The need for and change in resources was also challenging. The organization used an internal labor pool, and a large manufacturing company in the community provided an increased supply of basic resources. However, it's valuable to investigate creative staffing models and to use available resources in innovative ways. Also, education on PPE is necessary. In a study of pediatric nurses during the COVID-19 pandemic, Zheng found that having adequate PPE predicted less anxiety and depression.^29^ Leng and colleagues found that for nurses working during the pandemic in China, major sources of stress were concerns about PPE shortage and use.^30^ A study of nurses in Canada found that higher ratings of emotional distress were associated with negative ratings of workplace relations, organizational support and preparedness, workplace safety, and access to supplies.^12^

**Theme five: Emotional experience (stressful/scary)**. The emotional experiences of the nurses in this study were described as “stressful” and “scary.” Although there were resources available, it's crucial to make sure that nurses are aware of these resources. Spiritual care services, which could have been helpful to both patients and staff, weren't available during the beginning of the pandemic. Suggestions included the use of support groups during surges at the hospital but also continually thereafter. Many nurses had high levels of uncertainty, especially related to not knowing if or when they'd be caring for patients with COVID-19 and where they'd be working. Finding opportunities for everyone to contribute however they can is important, along with supporting nurses working in all areas, not only those working in acute care or with patients with COVID-19.

Similar emotional experiences related to COVID-19 have been reported by nurses from many countries. In a systematic review of qualitative studies of nurses' experiences working in hospitals during a respiratory pandemic in Spain, Fernandez and colleagues found fear and frustration were common in critical care nurses and led to emotional lability.^31^ These nurses required support both during and after the pandemic due to the physical and emotional impact. Halcomb and colleagues found that nurses in Australia identified concern for their mental health during and following the pandemic.^26^ Ardebili, in a qualitative study of healthcare providers in Iran, found that mental health deteriorated in stages as the pandemic unfolded.^32^ In the US, several studies have documented the emotional distress experienced by nurses.^3^,^12^,^22^

**Question two: Improvement strategies**. Improvement strategies were derived from responses to the second question. Participants referred to many strategies already in place, but data from the study provide additional strategies for improvement. Recommendations were made to develop strategies for decision-making, support, visibility of leaders, and communication. It's also important to design strategies that are user-friendly and efficient. It was clear that the nurses responding to the survey had ideas for how things could be improved and how they could be included in decision-making for improvement strategies. Table 3 identifies areas for improvement, noting the strategies that were already in place and possible strategies to implement as derived from the study.

**Table 3:** Improvement strategies

Improvement strategy areas	Strategies in place	Possible improvement strategies
Nurse input/presence in decision-making	Management rounds	Include staff representation in decision-making.
Preparedness and training	PPE video Policy updates Electronic tablets Scheduled times to communicate with family Links to procedures, policies, and more Refresher courses (EHR) Links (CDC, ANA, resources)	Use simulation, especially skills acquisition. Explain policy changes with reasons for change and in a format that's easy and quick to read. Develop creative strategies to communicate with families (individualized). Support family and staff with the transition from no visitors to visitors. Provide support to deal with fear/paranoia.
Staff support	Virtual meetings (caring for everyone)	Increase knowledge and awareness. Continue supportive measures, such as support groups, childcare, and spiritual care. Provide support for staff pulled to different floors to address issues such as uncertainty about where they'll be working and working in unfamiliar areas. Provide opportunities for everyone to contribute where they can and feel comfortable. Ensure everyone has the resources (internet) and support needed for their roles.
Visibility of management		Increase the presence of upper leadership (see a face). Make sure they're present for different shifts. Increase awareness about what leadership is doing when they're present.
Consistent, accessible, relevant, prompt communication	Daily emails Daily huddles Weekly updates by the CEO Visitor policy scripting Internal labor pool (such as retired, not working) COVID toolbox of resources COVID special alert emails 1-2 times per week	Streamline communication and find appropriate means of communication for clinical nurses, especially on off-shifts (nights). Increase use of pop-ups in the EHR. Use daily huddles for information sharing (including huddle board) and archive information for staff. Communicate about ongoing work related to staffing. Clarify staffing ratios.
COVID-19 practice demands	Internal labor pool Supply through business partners and other businesses	Implement creative staffing models. Provide education on different types of PPE. Make creative use of community resources.
Compensation	PTO benefit	Clarify PTO benefit use. Facilitate ability to use PTO.

## Implications for nurse leaders

Perceptions of leadership differed based on the nurse's role, suggesting a “one size fits all” approach may not be effective, and leaders should identify the needs of specific groups. However, regardless of the group, participants emphasized the importance of communication that was timely, focused for specific groups, and easily accessible. Nurse leaders had to make many decisions, and survey respondents suggested input and presence of staff in decision-making. Another recommendation was to balance the needs of nurses and patients using various support strategies, including increased visibility of leadership and additional resources, such as staff and education. At this agency, leaders have focused on communicating face-to-face when appropriate and including information relevant to specific groups.

Rapid practice changes required nurses to do things differently, so it was critical to ensure that nurses had the knowledge and skills to care for patients. When asked to provide care on units or for patients they weren't familiar with, nurses needed resources, including education and emotional support. The complexity of providing care for patients with COVID-19 calls for efficient and effective education. As the pandemic has progressed, the agency has developed methods of providing needed information and education, including regular updates using various communication methods.

The provision of resources was vital and included PPE, needed equipment, and adequate staffing to care for patients. Nurse leaders can facilitate the ease with which these resources are accessed and implement creative staffing models that work with the strengths of nurses and units.

The emotional experience of nurses was a dominant theme. Nurse leaders need to recognize the emotional toll on nurses working during the pandemic. Nurse leaders should educate themselves on trauma and make resources available to nurses, such as support groups, time off, and child-care assistance. Provision 5 of the ANA Code of Ethics emphasizes the responsibility of promoting the health and safety of self.^33^ In addition to providing staff members with opportunities for self-care, nurse leaders need to practice self-care as well. A study that included nurses in management roles found that one of the many challenges they experienced during the pandemic was providing support for everyone on their team while trying to strengthen their role.^25^

## Improving nurse experience

Findings from this study provide direction for nurse leaders and are consistent with other studies conducted during previous pandemics and during the COVID-19 pandemic. Stress is prevalent and is related to the work of caring for patients with COVID-19 and nurses' concerns for their own health and the health of their family members. This patient population requires complex care with frequent protocol changes, resulting in mental and physical stress on caregivers. Findings from this study provide suggestions from nurses working at the point of care. This study delineated the importance of leadership support related to both communication and developing relationships with staff. The amount of physical and psychological energy it takes to care for patients was also evident, along with the complexities of providing care when navigating changing knowledge and needs. The need for resources, both physical and from colleagues, was also evident. Overall, the emotional experience of providing care was apparent and described as stressful and scary. Nurse involvement in improving communication and providing support, team building, and education is important to enhance the experience of nurses and promote quality care. Based on the findings of this study, the organization has made changes to improve communication and provide more opportunities for nurses to be involved in making decisions.

## INSTRUCTIONS Nurse experience during the COVID-19 pandemic: Implications for nurse leaders

### TEST INSTRUCTIONS

- Read the article. The test for this nursing continuing professional development (NCPD) activity is to be taken online at www.NursingCenter.com/CE.
- You'll need to create an account (it's free!) and log in to access My Planner before taking online tests. Your planner will keep track of all your Lippincott Professional Development online NCPD activities for you.
- There's only one correct answer for each question. A passing score for this test is 7 correct answers. If you pass, you can print your certificate of earned contact hours and access the answer key. If you fail, you have the option of taking the test again at no additional cost.
- For questions, contact Lippincott Professional Development: 1-800-787-8985.
- Registration deadline is **March 7, 2025**.

### PROVIDER ACCREDITATION

Lippincott Professional Development will award 2.0 contact hours for this nursing continuing professional development activity.

Lippincott Professional Development is accredited as a provider of nursing continuing professional development by the American Nurses Credentialing Center's Commission on Accreditation.

This activity is also provider approved by the California Board of Registered Nursing, Provider Number CEP 11749 for 2.0 contact hours. Lippincott Professional Development is also an approved provider of continuing nursing education by the District of Columbia, Georgia, Florida, New Mexico, South Carolina, and West Virginia, CE Broker #50-1223. Your certificate is valid in all states.

Payment: The registration fee for this test is $21.95.
